# An Investigation of Vocal Tract Characteristics for Acoustic Discrimination of Pathological Voices

**DOI:** 10.1155/2013/758731

**Published:** 2013-10-31

**Authors:** Jung-Won Lee, Hong-Goo Kang, Jeung-Yoon Choi, Young-Ik Son

**Affiliations:** ^1^Department of Electrical and Electronic Engineering, Yonsei University, 134 Shinchon-dong, Seodaemun-gu, Seoul 120-749, Republic of Korea; ^2^Research Laboratory of Electronics, Massachusetts Institute of Technology, 50 Vassar Street, Cambridge, MA 02139, USA; ^3^Department of Otorhinolaryngology—Head and Neck Surgery, Samsung Medical Center, Sungkyunkwan University School of Medicine, 50 Irwon-dong, Gangnam-gu, Seoul 137–710, Republic of Korea

## Abstract

This paper investigates the effectiveness of measures related to vocal tract characteristics in classifying normal and pathological speech. Unlike conventional approaches that mainly focus on features related to the vocal source, vocal tract characteristics are examined to determine if interaction effects between vocal folds and the vocal tract can be used to detect pathological speech. Especially, this paper examines features related to formant frequencies to see if vocal tract characteristics are affected by the nature of the vocal fold-related pathology. To test this hypothesis, stationary fragments of vowel /aa/ produced by 223 normal subjects, 472 vocal fold polyp subjects, and 195 unilateral vocal cord paralysis subjects are analyzed. Based on the acoustic-articulatory relationships, phonation for pathological subjects is found to be associated with measures correlated with a raised tongue body or an advanced tongue root. Vocal tract-related features are also found to be statistically significant from the Kruskal-Wallis test in distinguishing normal and pathological speech. Classification results demonstrate that combining the formant measurements with vocal fold-related features results in improved performance in differentiating vocal pathologies including vocal polyps and unilateral vocal cord paralysis, which suggests that measures related to vocal tract characteristics may provide additional information in diagnosing vocal disorders.

## 1. Introduction

It is very important to evaluate acoustical voice quality for the assessment of pathological voice. The assessment process can be classified into two approaches: perceptive and objective. The perceptive assessment process qualifies and quantifies voice pathologies by directly listening to the voice of a subject. It is performed by trained professionals who evaluate the voice characteristics on a grade scale. The Buffalo voice profile analysis (BVP), the Hammarberg scheme, the vocal profile analysis scheme (VPA), and the GRBAS scale are typical examples [1]. The perceptive assessment is the most practical method used to evaluate and clinically manage pathological speech.

On the other hand, the objective assessment process utilizes signal processing techniques for measuring acoustic features in the temporal or spectral domain. Typical features are fundamental frequency (F0), perturbation measures such as jitter (changes in pitch with time) and shimmer (changes in amplitude with time), and harmonics-to-noise ratio (HNR) [2–8]. Some studies have been conducted using Mel-frequency cepstral coefficients (MFCCs) and their derivatives, which are the most widely used measurements to represent the speech signal in statistical speech signal processing systems [9–12]. Since the objective assessment approach offers the advantages of being quantitative, cheap, fast, and comfortable for the subject, it can be an effective method for screening and early detection of voice disorders [4, 5, 9, 10].

In the clinical area, the focus has been on clinical judgments of vocal qualities, which have been commonly derived from subjective grading systems rather than from objective assessment tools. Assessment tools such as the multidimensional voice program (MDVP) mainly supply the measurements, which give information related to the vocal source [13]. However, the articulatory configuration in the vocal tract interacts with the articulation in the vocal folds [14]; therefore, additional vocal tract-related information is expected to assist in detecting the characteristics of the vocal folds, especially during phonation.

Appropriate measures for reflecting the nature of the vocal tract should allow a consistent interpretation of the resulting numerical values, both with regard to normal speech, marking the extremes of voice quality and to other laryngeal pathologies [15]. Although MFCCs have been widely used in speech signal processing systems, the problem of using this acoustic measure in the assessment of pathological voice quality is the difficulty of interpreting MFCCs in relation to laryngeal physiology. For this reason, the physical relevance of MFCCs to vocal fold pathologies has not been deeply examined. In the case of more direct measurements of the vocal tract, the first two formants of vowels, which reflect the vocal tract structure, are used in a study by Muhammad et al. [16]. However, since they used a limited number of pathological recordings, the results are statistically weak. To date, no studies of vocal tract measurements for objective assessment have been investigated with a large database for pathological voice classification.

The objective of this paper is to analyze the impact of vocal tract information to discriminate normal and pathological voices. The vocal tract information is represented by formant frequencies (resonating frequencies of the vocal tract) and their variation in the temporal domain [17]. This paper first examines formant measurements using the Kruskal-Wallis test to assess their statistical significance, and results show that measurements for vocal tract-related features are significant for classifying normal and pathological speech. The changes of formant frequencies for pathological subjects are also analyzed based on the acoustic-articulatory relationships, indicating that their phonation is associated with a raised tongue body or an advanced tongue root. This analysis is consistent with the results in the linguistic literature. 

The actual classification with a support vector machine (SVM) classifier is performed with a large database, consisting of over 100 normal and 600 pathological subjects. The pathological subjects are comprised of subjects with vocal fold polyp and unilateral vocal cord paralysis (VCP) [18–20]. Due to the inherent differences in the speech production systems of female and male subjects, it is appropriate to deal with pathological speech classification separately for each gender [21]. Classification results verify that vocal tract-related features are useful in discriminating normal and pathological voices. The best performance can be obtained when the vocal tract and vocal fold-related features are combined. Finally, compared to results obtained with only vocal fold-related features such as F0, jitter, shimmer, and HNR, combining vocal tract measurements along with the vocal fold-related features reduces the relative equal error rate by 17.0%.

## 2. Materials and Methods

### 2.1. Materials

The voice recordings consist of utterances from pathological and normal speech collected by Samsung Medical Center, Seoul, Korea. The database contains phonation of the vowel /aa/, along with readings of a passage in Korean, recorded by 472 vocal fold polyp (232 females, 240 males), 195 unilateral VCP (106 females, 89 males), and 223 normal (99 females, 124 males) subjects (see Table 1). The subjects' ages ranged from 20 to 51 years old. The data samples were recorded in different sessions in a sound-treated booth, using a standardized recording protocol. In this study, only the stable part of sustained phonation of the vowel /aa/ is used. The sampling frequency is downsampled to 16 kHz.

### 2.2. Observation

**Figure 1:**
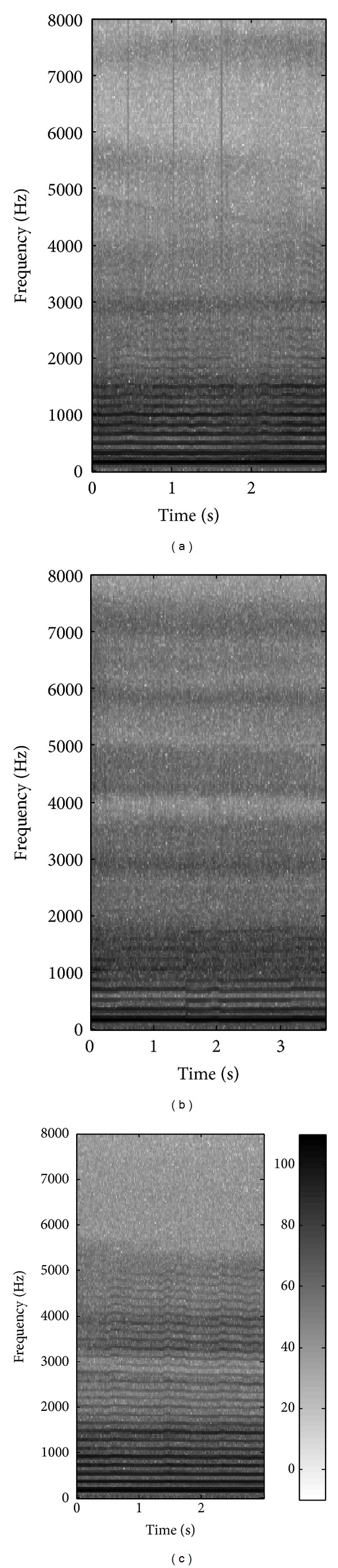
Spectrogram of example utterances from (a) vocal fold polyp, (b) unilateral VCP, and (c) normal female. Darker regions correspond to higher energies.

Figure 1(a) shows an example of a spectrogram obtained from an utterance from a subject with vocal fold polyp. Unlike the utterance from a normal subject, as shown in Figure 1(c), the vocal fold polyp utterance has pitch perturbation, unclear harmonics, turbulent noise, and voice breaks.  Figure 1(b) shows the spectrogram of an utterance from a subject with unilateral VCP. Only the first few harmonics are apparent, and the noise component increases in the high frequencies, especially over 3000 Hz.

To represent vocal tract-related spectral shape, an average autoregressive (AR) spectrum and the spectral difference between normal and pathological subjects are plotted. The order of coefficients was set to 16, which implies 8 maximal resonances of the vocal tract cavity. The average AR spectra obtained from /aa/ utterances for each subject were first normalized to the strongest peak between 500 and 1100 Hz to ensure that data across subjects were comparable to each other regardless of their absolute power.

**Figure 2:**
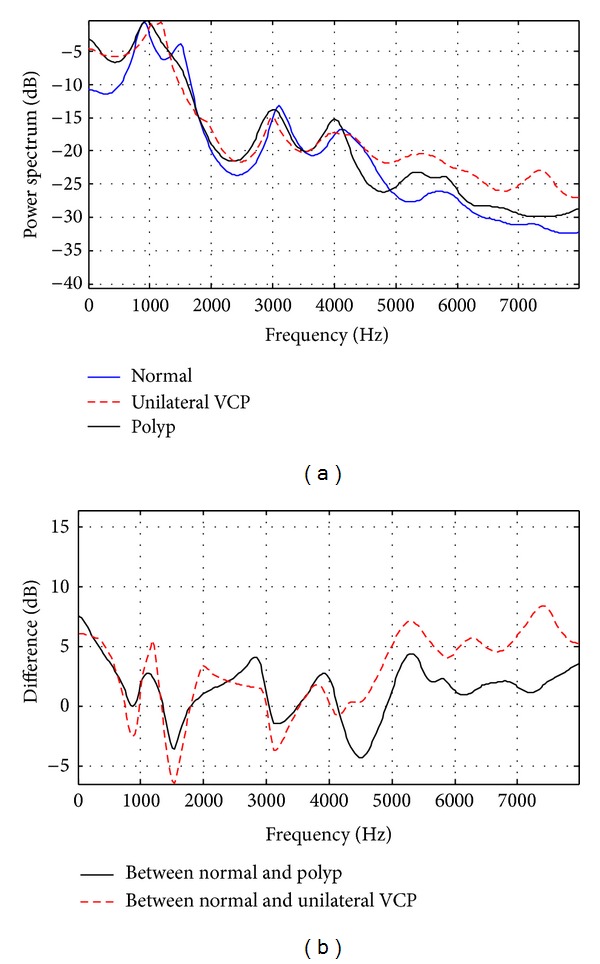
(a) Average linear prediction (LP) spectrum and (b) spectral difference of normal and pathological female subjects.

**Figure 3:**
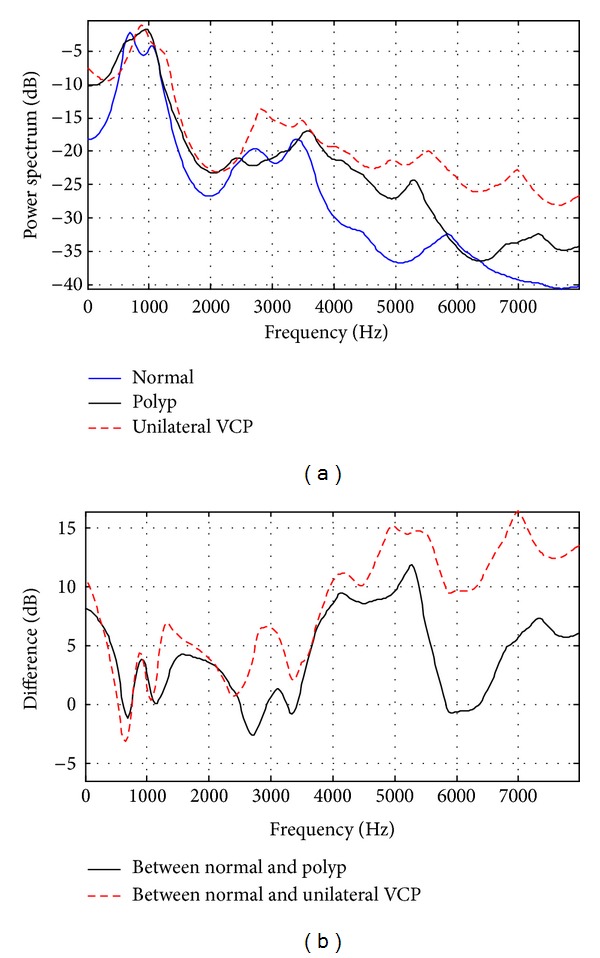
(a) Average linear prediction (LP) spectrum and (b) spectral difference of normal and pathological male subjects.

Figures 2 and 3 show the average AR spectra and spectral differences, using five utterances from each group. They show that the overall shape of the AR spectrum for normal subjects differs from that for both pathological groups, especially in the region below 500 Hz and over 4000 Hz. It is generally known that the spectral differences in those regions between normal and pathological groups are results of differences in manipulation of the laryngeal structures that are employed in phonation during vocalic segments [22–25]. The relative amplitudes of the harmonics are affected by the shape of the glottal pulse. The slower the glottal pulse returns to zero after the peak, the larger the amplitude of the first harmonic. Breathy phonation, such as in pathological voice, is characterized by a glottal source with an increased open quotient and results in a change of high amplitude in a low frequency band [23–25]. Also, breathy phonation by a glottal source often masks higher harmonics with aspiration noise [22, 23]. These observations can be also identified in Figure 1.

Another observation is that the degree of difference in the AR spectrum between normal and unilateral VCP subjects is bigger than that between normal and vocal fold polyp subjects. In order to measure the distance of two average LP spectra, a log spectral distance measure *D* is introduced. Consider

(1) $$\begin{array}{c} D=\sqrt{\frac{1}{F_{s}}\int_{0}^{F_{s}}{\left[10\log_{10}\left(P\left(f\right)\right)-10\log_{10}\left(\hat{P}\left(f\right)\right)\right]}^{2}df}, \end{array}$$

where *F*_*s*_ is the sampling frequency in hertz, and *P*(*f*) and $\hat{P}(f)$ are the average AR power spectra of a specific pathological group and the normal group, respectively [26]. The results of polyp and unilateral VCP groups are *D* = 2.7 and 4.3 for females and *D* = 5.6 and 9.7 for males, respectively. This indicates that the discrimination of pathological voice for unilateral VCP subjects is easier than the one for vocal fold polyp subjects. It is also expected that male subjects are more easily identified than female subjects when using vocal tract characteristics.

Another observation from the average AR spectrum is that the positions of peaks in the middle frequency band are not similar to one another. The resonance frequencies of the vocal tract, or the formants, correspond approximately to the peaks of the spectrum of the vocal tract response [17]. Formant frequencies for phonation of the vowel /aa/ are known to be around 900 and 700 Hz for the first formant (F1), around 1400 and 1200 Hz the for second formant (F2), and around 2800 and 2500 Hz for the third formant (F3), for female and male speakers, respectively [14]. Males usually have lower formant frequencies than that of females.

These observations imply that vocal tract characteristics may provide the capability of classifying normal/pathological subjects although pathological subjects have disorders which are directly related to the vocal folds.

### 2.3. Acoustic Measures from Speech Stimuli

#### 2.3.1. Vocal Tract-Related Features

Among many vocal tract-related features, this paper adopts formant frequencies to represent the effects of vocal tract configurations, as they reflect the resonance frequencies of vocal tract. Based on the results in Section 2.2 that the mean and variance of formant frequencies between normal and pathological speech are different, the mean and standard deviation of F1, F2, and F3 are extracted, which are referred to as *static features*. In addition, the temporal variability, that is, *dynamic features*, can be an important characteristic to discriminate pathological speech from normal speech. Dynamic features can be obtained by calculating time derivatives to the basic static features (referred to as delta features). The delta features are computed using the following formula [27]:

(2) $$\begin{array}{c} d_{n}=\frac{\sum_{\theta =1}^{\alpha }\theta \left(F_{n+\theta }-F_{n-\theta }\right)}{2\sum_{\theta =1}^{\alpha }\theta^{2}}, \end{array}$$

where *d*_*n*_ is a delta feature at frame *n* computed in terms of the corresponding static features *F*_*n*+*θ*_ − *F*_*n*−*θ*_. The value *α* is set to two in our experiments.

#### 2.3.2. Vocal Source-Related Features

Pathologies such as vocal fold polyp and unilateral VCP affect the vocal fold or other components of the voicing system. They result in irregular vibration and incomplete closure of the vocal folds in glottal cycles. The acoustic signal reflects these changes in the vocal folds. Clinically, acoustic measures such as the fundamental frequency and amplitude perturbation coefficients (jitter and shimmer, resp.) and harmonics-to-noise ratio (HNR) are typically used to characterize pathological voice. In this paper, the following four measures are also used as conventional measures of vocal quality: F0, jitter, shimmer, and HNR.

(1) F0: average value of all extracted period-to-period fundamental frequency values in sustained vowel is measured. Consider
  (3) $$\begin{array}{c} F_{\text{mean}}=\frac{1}{N_{\text{tot}}}\sum_{i=1}^{N_{\text{tot}}}F_{i}, \end{array}$$

where *F*_*i*_ is the fundamental frequency F0 in cycle *i*, and *N*_tot_ is the number of frames in the utterance. The average value of F0 is expected to be similar between normal and pathological subjects, but the standard deviation of F0 for pathology is expected to be larger than that for normal speech.

(2) Jitter: jitter refers to the variability of F0, which is calculated by average absolute difference between consecutive periods, divided by the average period [3]. Consider
  (4) $$\begin{array}{c} \text{jitter}\left(l\right)=\frac{\left(1/\left(N-1\right)\right)\sum_{i=1}^{N-1}\left|T_{i,l}-T_{i+1,l}\right|}{\left(1/N\right)\sum_{i=1}^{N}T_{i,l}}, \end{array}$$

where *T* are the extracted F0 period lengths, *N* is the number of extracted F0 to calculate jitter, and *l* is the frame index. The average value of jitter obtained in the entire utterance is used. Jitter for pathological utterances is expected to be larger than that for normal subjects.

(3) Shimmer: shimmer refers to the variability of the peak-to-peak amplitude, and relative shimmer is calculated by the average absolute difference between the amplitudes of consecutive periods, divided by the average amplitude [3]. Consider
  (5) $$\begin{array}{c} \text{shimmer}\left(l\right)=\frac{\left(1/\left(N-1\right)\right)\sum_{i=1}^{N-1}\left|A_{i,l}-A_{i+1,l}\right|}{\left(1/N\right)\sum_{i=1}^{N}A_{i,l}}, \end{array}$$

where *A* is the extracted peak-to-peak amplitude. The average value of shimmer obtained in the entire utterance is used. Shimmer values for pathological utterances are expected to be larger than that for normal subjects, similar to the case of jitter.

(4) HNR: HNR employed here is calculated based on the residuals obtained by long-term predictive analysis [7, 8]. HNR is defined as the energy ratio between the periodic and aperiodic components as follows:
  (6) $$\begin{array}{c} \text{HNR}\left(l\right)=20\log\left(\frac{\sum_{m=1}^{M_{j}}\left|\left|S\left(m,l\right)\right|-\left|N\left(m,l\right)\right|\right|}{\sum_{m=1}^{M_{j}}\left|N\left(m,l\right)\right|}\right), \end{array}$$

where *S*(*m*, *l*) and *N*(*m*, *l*) are the short-time Fourier transforms of target signal and aperiodic components, respectively. The terms *l*, *m*, and *M*_*j*_ are the frame index, frequency bin index, and number of frequency bins, respectively. Aperiodic components *N*(*m*, *l*) can be considered as the residuals of long-term predictive analysis. The current analysis frame of length *L* is predicted by a lagged analysis frame of the same length such that

(7) $$\begin{array}{c} \hat{s}\left(k\right)=\beta s\left(k-T\right), \end{array}$$

where *s*(*k*) is the current target speech sample, *T* is the prediction lag with −*T*_max⁡_ ≤ *T* ≤ −*T*_min⁡_ and *T*_min⁡_ ≤ *T* ≤ *T*_max⁡_, and *β* is the long-term prediction coefficient. *T*_max⁡_ and *T*_min⁡_ are fixed to 25 ms and 2.5 ms, respectively. The optimal long-term prediction coefficient is derived by minimizing the prediction error energy *E*, that is,

(8) $$\begin{array}{c} E=\sum_{k=0}^{L-1}e^{2}\left(k\right)=\sum_{k=0}^{L-1}{\left[s\left(k\right)-\beta s\left(k-T\right)\right]}^{2}, \end{array}$$

which yields that

(9) $$\begin{array}{c} \beta =\frac{\sum_{k=0}^{L-1}s\left(k\right)s\left(k-T\right)}{\sqrt{\sum_{k=0}^{L-1}s^{2}\left(k\right)\sum_{k=0}^{L-1}s^{2}\left(k-T\right)}}.\; \end{array}$$

*β* is bounded to be equal to or less than 1. The optimum value is the lag for which the prediction error energy becomes minimum; that is,

(10) $$\begin{array}{c} T_{\text{opt}}=\arg\;\underset{T}{\min}\left\{\sum_{k=0}^{L-1}{\left[s\left(k\right)-\beta s\left(k-T\right)\right]}^{2}\right\}. \end{array}$$

The instantaneous value of the prediction error (residual signal) is calculated as follows:

(11) $$\begin{array}{c} e\left(k\right)=s\left(k\right)-\beta s\left(k-T_{\text{opt}}\right). \end{array}$$

The short-time Fourier transform of *e*(*k*) becomes *N*(*m*, *l*). In this paper, *s*(*k*) is the linear predictive residual signal, and the average HNRs obtained from the entire utterance are used. HNR measures for normal subjects are expected to be larger than that for pathological subjects.

### 2.4. Experimental Setup

In order to provide reliable pitch information, an adaptive time-domain pitch-synchronous method used in the MDVP manual was employed [13, 28]. Using the period-to-period pitch obtained, features related to F0, jitter, and shimmer are calculated. Formant frequencies were extracted every 10 ms, using the Praat software [29]. For HNR, aperiodic components were calculated from speech signals at every 2.5 ms, using a 5 ms Hanning window.

The extracted features are concatenated as a vector, called the input vector, and then a statistical model is built. In modeling of distributions for normal or pathological subjects, *m*-fold cross validation is used to reduce the influence of training tokens [30]. In this paper, each group is divided into ten sections. Discrimination between normal and pathological subjects is conducted using SVM with a radial basis function kernel.

In order to evaluate the performance of the discrimination between normal and pathological subjects, detection error tradeoff (DET) and equal error rate (EER), which is the rate at which both missed detection and false alarm error are equal, are used. The DET curve and EER have been used widely for the assessment of detection performance in various tasks, such as speaker identification [31]. The distance of SVM output is used to obtain EER. For comparison of results, relative error improvement is given as

(12) $$\begin{array}{c} \text{relative}\;\;\text{improvement}\;\left(\%\right)=\frac{{\text{EER}}_{b}-{\text{EER}}_{i}}{{\text{EER}}_{b}}\times 100\%, \end{array}$$

where EER_*b*_ and EER_*i*_ are base EER and improved EER, respectively. For performance evaluation, we deal with pathological speech detection separately for each gender [21].

## 3. Results and Discussion

### 3.1. Evaluation of Formant Frequencies

**Figure 4:**
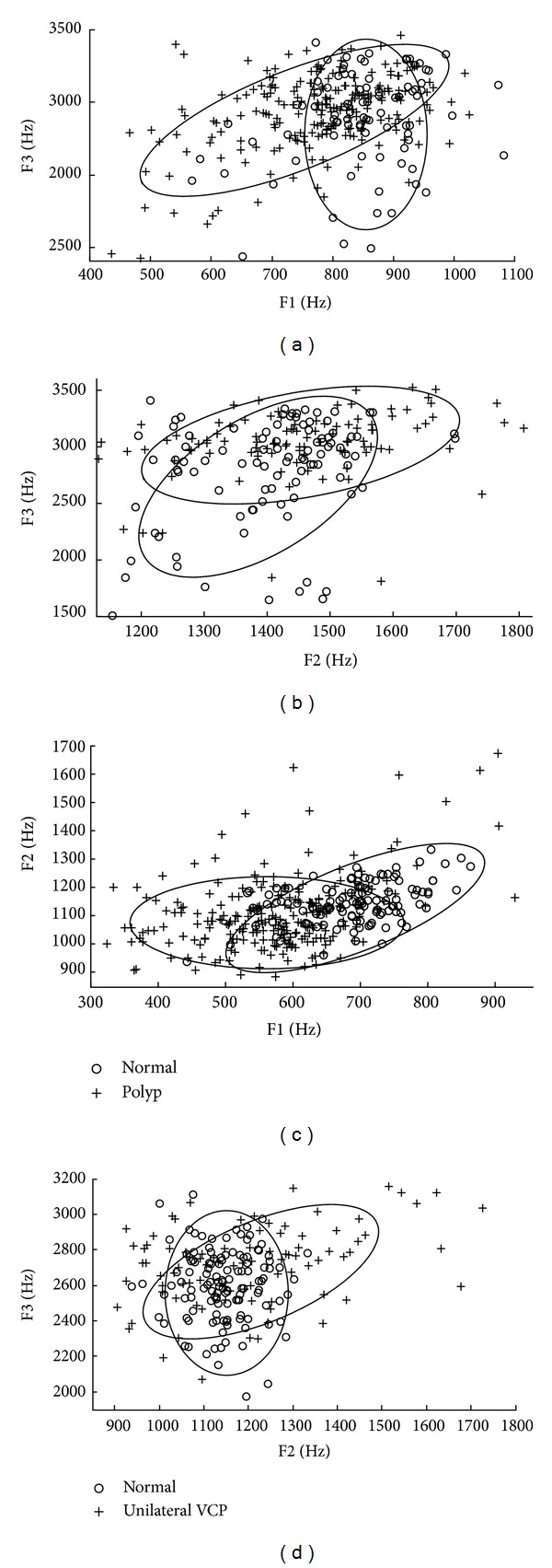
Distribution of average formant frequency for each normal versus polyp (left) and normal versus unilateral VCP (right) subjects for females ((a) and (b)) and males ((c) and (d)). Two discriminatory formants from among F1, F2, and F3 are selected for each plot. Regions for distributions are marked with ellipses. For the case of female vocal fold polyp data, F3 mean was used in plotting the figure although its *P* value is larger than 0.05, because only F1 mean was found to be a significant feature from among three formant means.

The formant measurements that represent vocal tract characteristics are first examined using the Kruskal-Wallis test between normal and vocal fold polyp data, and between normal and unilateral VCP data. Tables 2 and 3 show results of statistical feature analysis using static features in normal, vocal fold polyp, and unilateral VCP subjects for female and male subjects, respectively. One-way analysis is performed for each of the static features, and significant features with *P* < 0.05 are found.  Figure 4 shows the distribution of average formant frequencies for each subject. 

As for the average formants in Tables 2 and 3, they are significant in discriminating normal and pathological data. Average F1 is significant in discriminating normal and vocal fold polyp subjects. Vocal fold polyp subjects have average F1 of 763 Hz and 560 Hz, which are 81 Hz and 116 Hz smaller than that of normal subjects, for female and male speakers, respectively. In the discrimination of normal and unilateral VCP subjects, F2 mean and F3 mean are significant for both genders. Unilateral VCP subjects have average F2 of 1436 Hz and 1210 Hz, which are 43 Hz and 65 Hz higher than average F2 of normal subjects, and average F3 of 2994 Hz and 2716 Hz, which are 232 Hz and 115 Hz higher than average F3 of normal subjects, for female and male speakers, respectively. 

By changing the vocal tract shape, different resonating frequencies (formants) are produced. It is known that the frequencies of the first two formants, F1 and F2, are related to dimensions of vowel articulation [14]. The frequency of F1 is inversely related to tongue height, and the frequency of F2 is related to tongue advancement. Based on this fact, the observed lower F1 of vocal polyp subjects implies that the tongue occupies a higher position during phonation. Also, the higher F2 of unilateral VCP subjects may indicate that the tongue moves to a more anterior position during phonation. In accordance with the fact that breathiness is a very common symptom of pathological speech [32], our results are consistent with the results in the literature that breathy phonation is associated with a raised tongue body or an advanced tongue root across a variety of languages [33, 34]. This inferred difference of the position of the tongue compared to normal subjects suggests that the shape of the vocal tract is changed during phonation for pathological subjects. 

As for the standard deviation of the formants in Tables 2 and 3, it shows that all measurements except that of F3 in females are significant for the discrimination of normal and vocal fold polyp or unilateral VCP subjects. Furthermore, all measurements of pathology which are significant have higher standard deviation values than that of normal subjects. The high values of standard deviation indicate a more unstable vocal tract configuration during phonation for subjects with voice disorders. Based on the fact that unilateral VCP subjects have higher standard deviation than subjects with vocal fold polyp, and our observation that log spectral distance between normal and unilateral VCP subjects is higher than that between normal and vocal fold polyp subjects, as shown in Section 2.2, the vocal tract configuration of unilateral VCP subjects is inferred to be more unstable than that of vocal fold polyp subjects during phonation.

As for the dynamic features in Tables 4 and 5, similar characteristics can be observed. All standard deviation measurements except that of F3 for females between normal and unilateral VCP subjects are significant for the discrimination of normal and pathological speech in both genders, while most of the average values of dynamic features are not significant. For significant features, the mean of each formant standard deviation for unilateral VCP subjects is higher than that of the vocal fold polyp subjects. It indicates that vocal tract for pathology, especially unilateral VCP, is unstable compared to that for normal subjects.

**Figure 5:**
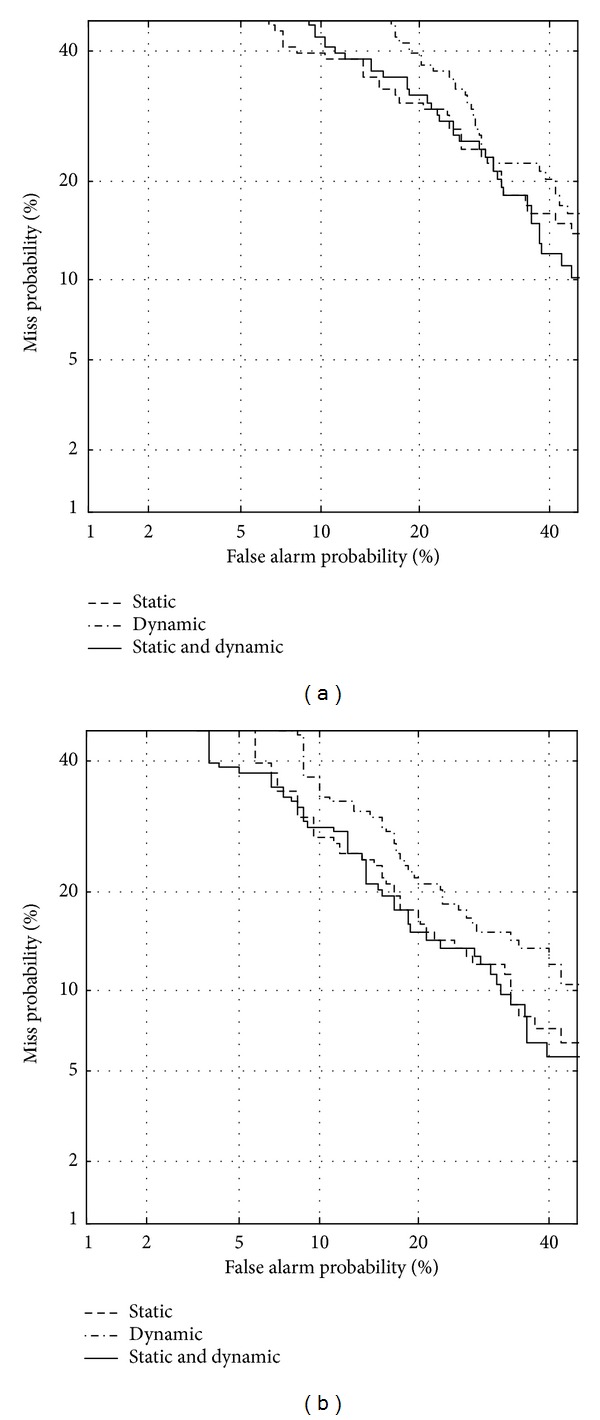
DET curve using static and dynamic features for discriminating between normal and vocal fold polyp subjects, for (a) females and (b) males.

**Figure 6:**
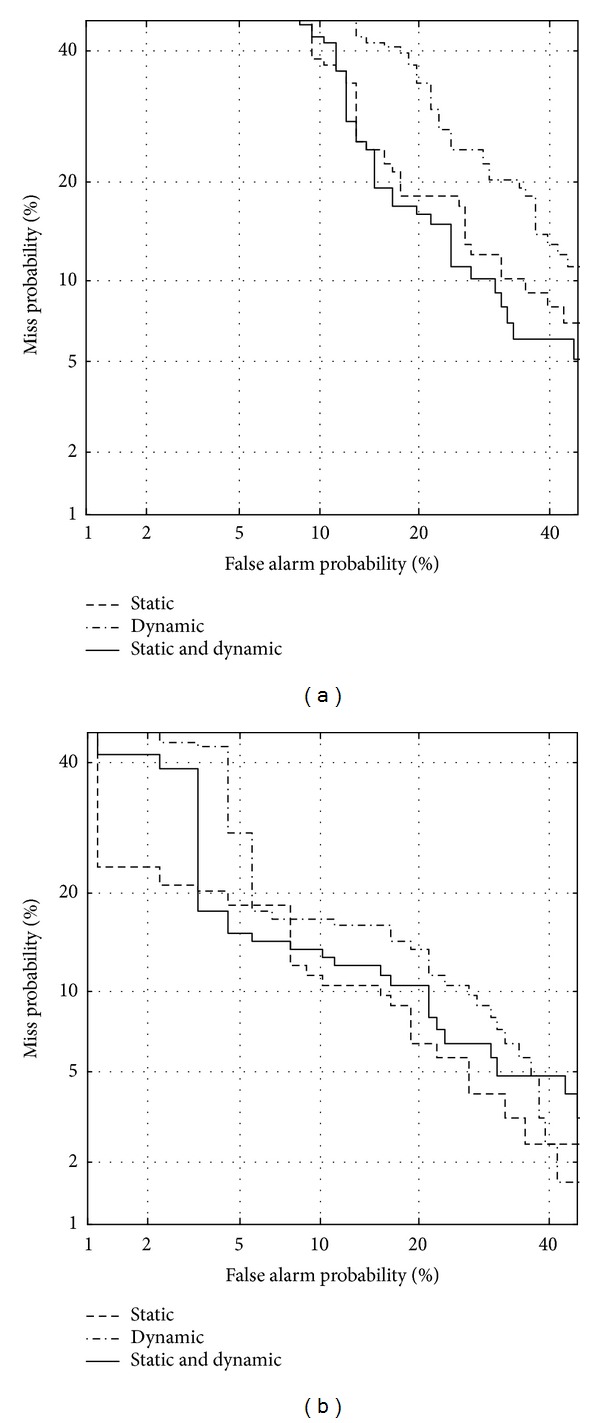
DET curve using static and dynamic features for discriminating between normal and unilateral VCP subjects, for (a) females and (b) males.

Figures 5 and 6 show DET curves to verify the classification performance using static and dynamic features between normal and vocal fold polyp subjects, and between normal and unilateral VCP subjects, respectively.  Table 6 shows EER for each case depicted in Figures 5 and 6. Both static and dynamic features are significant to classify pathological subjects from normal ones. Although dynamic features show lower performance than that of static features, results indicate that the unstable measurements for vocal tract characteristics are important in classifying normal and pathological speech. By combining static with dynamic features, performance is further enhanced for female subjects. Overall results indicate that vocal tract-related features are capable of discriminating normal and pathological speech although pathologies are situated at the vocal folds. This corresponds to the knowledge in the literature that the functioning of the vocal folds is not independent of the vocal tract [14]. Titze and Story [35] point out that the epilarynx (the narrow portion of the pharynx located directly superior to the vocal folds) is shaped in such a way that it enhances the interactions between the source and vocal tract, suggesting that pathology at the vocal folds affects the vocal tract shape.

In the case of unilateral VCP subjects, discrimination shows better performance than that for the vocal fold polyp subjects. Considering the observation seen in Section 2.2, and statistical analysis above, it is concluded that vocal tract modification for unilateral VCP subjects is greater than that for vocal fold polyp subjects. 

When it comes to gender, EERs for males are lower than those for females. The reason can be inferred to be that pathological male subjects attempt to change the vocal tract shape more than the female subjects do. Further research and experiments are needed to see if this conclusion is correct.

### 3.2. Combining Formant Features with Vocal Fold-Related Features

**Figure 7:**
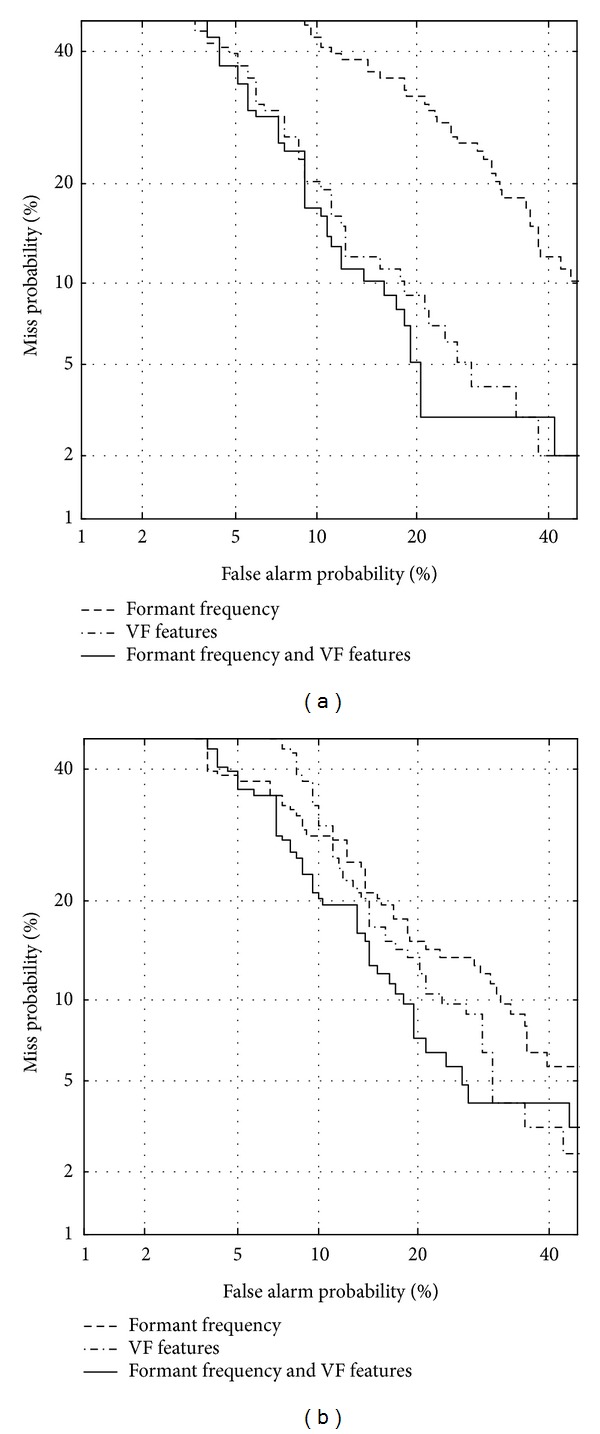
DET curve using formants and vocal fold-related features for discriminating between normal and vocal fold polyp subjects, for (a) females and (b) males.

**Figure 8:**
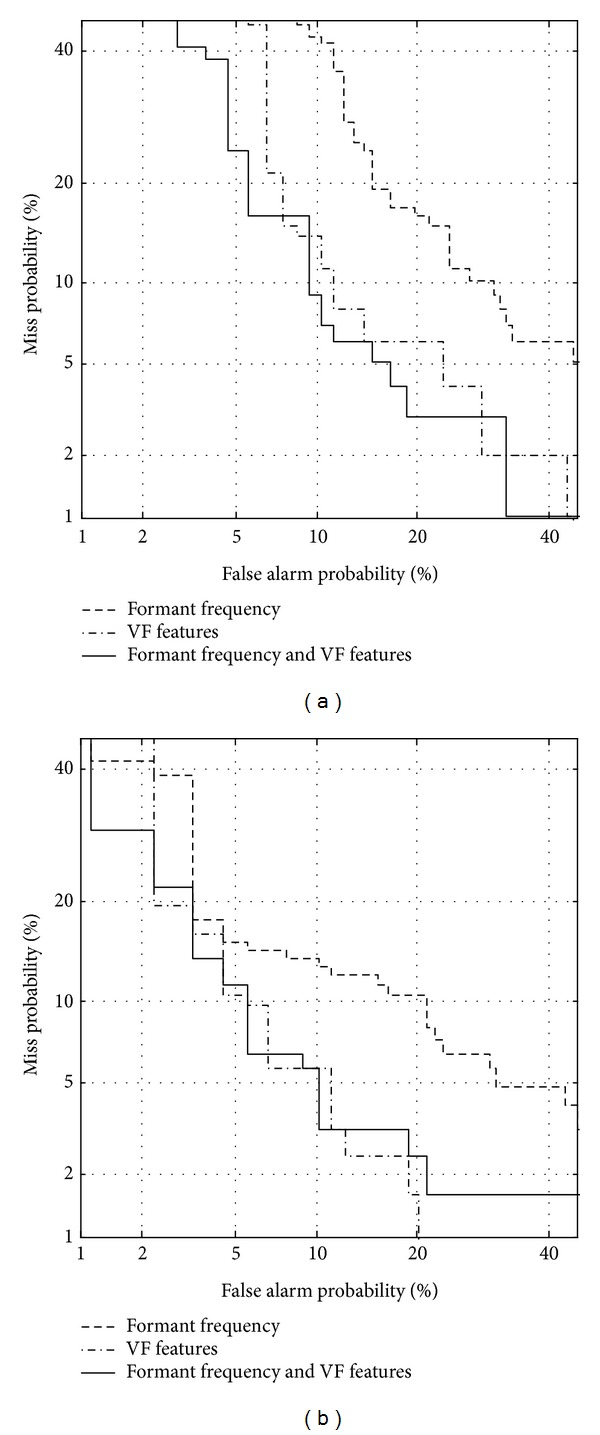
DET curve using formants and vocal fold-related features for distinguishing between normal and unilateral VCP subjects, for (a) females and (b) males.

By combining formant features (including static and dynamic features) with vocal fold-related features, a classification test is performed. Figures 7 and 8 show DET curves between normal and vocal fold polyp or unilateral VCP subjects, respectively.  Table 7 shows EER for each case depicted in Figures 7 and 8. The results show that the performance from combining vocal fold features with formant features is better than that with only vocal fold features except for unilateral VCP males. For unilateral VCP male subjects, it seems that classification performance is saturated. Relative errors are improved by 1.6%, 9.4%, 17.0%, and 0%, respectively. This implies that in order to classify pathological subjects such as vocal fold polyp and unilateral VCP from normal subjects, information related to both vocal tract and vocal fold is needed.

Unilateral VCP speech is well identified from normal speech compared to vocal fold polyp, when any feature group (vocal tract, vocal fold, and both) is used for each gender. The results imply that speech of unilateral VCP subjects may be more different from normal speech compared to that of vocal fold polyp subjects.

## 4. Conclusions

In this study, the importance of vocal tract characteristics for acoustic discrimination of pathological voices in vocal polyp and unilateral vocal cord paralysis has been analyzed. In the clinical field, objective assessment tools for vocal fold pathologies have been usually supplemented with perceptual judgments, as factors separated from vocal fold measurements have not been readily available. 

By measuring the statistical significance of formant measurements, which is directly related to the vocal tract, it is observed that the vocal tract characteristics may also be indicative of vocal fold-related pathology. Classification systems using formant measurements (including static and dynamic features) yield consistent discrimination between normal and pathological speech. Experimental results also show that measurements of the vocal tract combined with vocal fold-related features consistently outperform the case of only using vocal fold-related features, suggesting that these features provide additional information to vocal fold-related features.

In the gender-dependent experiments using, only formant measurements, EERs for male speakers are lower than those for female speakers. Further research and experiments are needed to understand why and how vocal fold pathologies lead to changes in vocal tract configuration during phonation, for example, reasons related to physiological or psychological compensations, and so forth.

In our study, classification is easier for unilateral VCP than for vocal fold polyp subjects. Future work relates to studying other types of pathological voices, and analysis of images or video signals displaying the articulatory organs may also be helpful.

## Figures and Tables

**Table 1:** Normal and pathological (vocal fold polyp and unilateral VCP) recordings.

	No. of females	No. of males	Total no.
Normal	99	124	223
Vocal fold polyp	232	240	472
Unilateral VCP	106	89	195

**Table 2:** Comparison of static features between the first three formants of the vowel /aa/ in normal, polyp, and unilateral VCP subjects for females. The term SD indicates standard deviation.

	F1 mean			F1 SD			F2 mean			F2 SD			F3 mean			F3 SD		
	Mean	SD	*P* value	Mean	SD	*P* value	Mean	SD	*P* value	Mean	SD	*P* value	Mean	SD	*P* value	Mean	SD	*P* value
Normal	844	103		66.6	73.1		1393	127		87.8	82.3		2762	481		240.6	250.4	
Polyp	763	134	0	99.4	72.3	0	1380	154	0.639	99.8	64.8	0.001	2894	326	0.128	237.6	229.0	0.139
Unilateral VCP	791	200	0.148	117.9	90.8	0	1436	169	0.027	113.3	105.3	0.003	2994	376	0	194.7	195.2	0.804

**Table 3:** Comparison of static features between the first three formants of the vowel /aa/ in normal, polyp, and unilateral VCP subjects for males. The term SD indicates standard deviation.

	F1 mean			F1 SD			F2 mean			F2 SD			F3 mean			F3 SD		
	Mean	SD	*P* value	Mean	SD	*P* value	Mean	SD	*P* value	Mean	SD	*P* value	Mean	SD	*P* value	Mean	SD	*P* value
Normal	676	79		23.8	23.6		1145	72		29.3	35.6		2601	202		66.9	58.9	
Polyp	560	108	0	63.5	58.6	0	1105	123	0	83.8	85.3	0	2577	237	0.194	120.2	102.5	0
Unilateral VCP	663	145	0.320	117.8	75.5	0	1210	201	0.048	156.2	135.4	0	2716	221	0	134.8	88.1	0

**Table 4:** Comparison of dynamic features between the first three formants of the vowel /aa/ in normal, polyp, and unilateral VCP subjects for females. The term SD indicates standard deviation.

	Delta F1 mean			Delta F1 SD			Delta F2 mean			Delta F2 SD			Delta F3 mean			Delta F3 SD		
	Mean	SD	*P* value	Mean	SD	*P* value	Mean	SD	*P* value	Mean	SD	*P* value	Mean	SD	*P* value	Mean	SD	*P* value
Normal	−0.03	1.15		18.5	22.5		−0.03	1.15		18.5	22.5		−0.03	1.15		18.5	22.5	
Polyp	−0.16	0.66	0.627	28.3	22.8	0	−0.16	0.66	0.627	28.3	22.8	0	−0.16	0.66	0.627	28.3	22.8	0
Unilateral VCP	−0.28	0.91	0.761	32.7	26.0	0	−0.28	0.91	0.761	32.7	26.0	0	−0.28	0.91	0.761	32.7	26.0	0

**Table 5:** Comparison of dynamic features between the first three formants of the vowel /aa/ in normal, polyp, and unilateral VCP subjects for males. The term SD indicates standard deviation.

	Delta F1 mean			Delta F1 SD			Delta F2 mean			Delta F2 SD			Delta F3 mean			Delta F3 SD		
	Mean	SD	*P* value	Mean	SD	*P* value	Mean	SD	*P* value	Mean	SD	*P* value	Mean	SD	*P* value	Mean	SD	*P* value
Normal	−0.13	0.45		4.9	7.1		0.03	0.66		6.5	10.9		−0.01	1.23		16.4	17.9	
Polyp	−0.08	0.50	0.515	16.4	18.3	0	0.03	0.52	0.305	23.7	27.3	0	0.09	0.75	0.736	36.2	32.6	0
Unilateral VCP	0.31	1.01	0	35.3	25.9	0	0.31	1.18	0.097	50.1	46.6	0	0.28	0.96	0.027	43.4	30.1	0

**Table 6:** Equal error rate (in %) using static and dynamic features related to formants.

	Static	Dynamic	Static + dynamic
Polyp			
Female	26.1	28.2	25.3
Male	17.8	20.9	17.8
Unilateral VCP			
Female	18.1	24.4	17.1
Male	10.3	15.9	12.2

**Table 7:** Equal error rate (in %) using formants and vocal fold-related features. The term VF indicates vocal fold-related features. Numbers in parentheses show relative error improvement in equal error rate (in %) compared to using only VF.

	Formants	VF	Formants + VF
Polyp			
Female	25.3	12.3	12.1 (1.6)
Male	17.8	16.0	14.5 (9.4)
Unilateral VCP			
Female	17.1	11.2	9.3 (17.0)
Male	12.2	6.6	6.6 (0)
